# Simultaneous signal optimization of refraction and attenuation in x‐ray grating interferometry: A case study for breast imaging

**DOI:** 10.1002/mp.70069

**Published:** 2025-10-09

**Authors:** Alexandre Pereira, Michał Rawlik, Simon Spindler, Stefanie Kaser, Dominik Etter, Gianluca Iori, Martin Stauber, Lucia Romano, Marco Stampanoni

**Affiliations:** ^1^ Institute for Biomedical Engineering ETH Zürich and University of Zürich Zürich Switzerland; ^2^ Swiss Light Source Paul Scherrer Institute Villigen Switzerland; ^3^ GratXray Villigen Switzerland

**Keywords:** breast imaging, grating interferometry, system design

## Abstract

**Background:**

X‐ray grating interferometry has emerged as a promising imaging technique for breast computed tomography (BCT), offering complementary contrast from refraction beyond conventional absorption imaging. Although it provides higher resolution, the required dose levels remain above those typically used for breast cancer diagnostic imaging.

**Purpose:**

This study aims to design an optimized grating interferometry‐based BCT system using a novel optimization metric that simultaneously maximizes signal strength from refraction and attenuation while considering grating fabrication and system constraints.

**Methods:**

A systematic grid search was conducted to identify system configurations that optimize contrast‐to‐noise ratio while accounting for dose efficiency in both attenuation and refraction. The optimized system was benchmarked against a commercial absorption‐based BCT system and a previously published grating interferometry design. Simulations were performed using in silico breast phantoms of varying diameters, incorporating realistic imaging conditions and noise modeling across dose levels from 1 to 100 mGy. Additionally, a signal fusion technique was applied to combine the two contrast channels.

**Results:**

Quantitative analysis demonstrates that refraction provides superior contrast for small malignancies, while absorption remains beneficial for larger lesions. Fusing both contrast channels enhances the contrast‐to‐noise ratio and improves resolution in in silico reconstructed images, outperforming conventional absorption‐based BCT at dose levels relevant for breast cancer diagnostic imaging.

**Conclusions:**

The findings suggest that an optimized grating interferometry‐based BCT system can improve image quality by effectively combining absorption and refraction signals. This work underscores the need to balance imaging performance with practical implementation constraints, offering a framework that may extend to other imaging applications beyond breast cancer detection.

## 1 INTRODUCTION

Breast imaging is a cornerstone of diagnostic radiology, employing various modalities to enhance the visualization and characterization of breast tissue. Mammography remains the most widely utilized technique;^1^ however, its limited soft‐tissue contrast and reliance on 2D projections pose challenges in accurately interpreting complex breast anatomy. These limitations can hinder the detection of subtle abnormalities. To overcome the issue of tissue superimposition, 3D imaging techniques such as digital breast tomosynthesis (DBT) have been introduced. DBT offers improved lesion conspicuity by providing quasi‐3D reconstructions, though its diagnostic performance shows only incremental gains over conventional mammography.^2^ Additionally, DBT still necessitates breast compression, which may affect patient comfort and image quality. Magnetic resonance imaging (MRI) presents an alternative with superior soft‐tissue contrast and the advantage of avoiding ionizing radiation. Despite its high sensitivity, MRI is constrained by lower spatial resolution, reduced specificity, prolonged acquisition times, and elevated costs, which limit its broader clinical adoption.^3^, ^4^

A technique that is increasingly being adopted in clinical practice is dedicated breast computed tomography (BCT), which addresses the limitations of both MRI and mammography. BCT provides a 3D visualization of the entire breast without the need for compression. Still, it suffers from poor soft‐tissue contrast, particularly when distinguishing tumors embedded in fibroglandular tissue, especially in dense breasts. As a result, the use of contrast‐enhancement agents is often necessary to improve lesion detectability.^5^

In recent years, x‐ray phase‐contrast (PC) imaging has emerged as a promising modality for breast imaging. Unlike conventional x‐ray techniques, PC imaging captures not only absorption but also refraction and small‐angle scattering effects induced by the tissue, thereby providing enhanced contrast and structural information. Several PC imaging approaches have been investigated for breast applications, including propagation‐based PC,^6^, ^7^ edge‐illumination,^8^ and grating interferometry (GI).^9^ Propagation‐based PC has demonstrated encouraging results at various synchrotron facilities when compared to traditional absorption‐based breast computed tomography (BCT).^6^, ^7^, ^10^ However, its reliance on high spatial coherence limits its feasibility for clinical implementation.

In laboratory settings, the advantages of PC over attenuation‐based imaging have primarily been demonstrated in high‐dose, high‐resolution regimes. Notably, GI has shown significant benefits in virtual histopathology.^11^, ^12^ For large field‐of‐view (FOV) systems, edge‐illumination has been explored for ex vivo tumor margin assessment.^13^ Within the context of GI, multiple studies have reported promising outcomes in PC mammography, particularly in the improved detection and characterization of microcalcifications.^14^, ^15^, ^16^, ^17^, ^18^ More recently, a GI‐based BCT system has been shown to achieve superior image quality–measured by contrast‐to‐noise ratio (CNR)—when PC and attenuation images are fused. However, this improvement comes at the cost of radiation doses approximately four times higher than those currently used in clinical practice.^19^ Reducing this dose remains a key challenge, with ongoing efforts focused on enhancing grating quality. Nonetheless, fabricating high‐performance gratings for high‐energy x‐ray applications while maintaining system sensitivity continues to pose significant technical hurdles.^20^

Motivated by the improved image quality by fusing the two contrast channels,^19^ a novel optimization metric is introduced, providing a general framework for optimizing a GI system by leveraging both the refraction and conventional attenuation signals simultaneously. Although demonstrated here in the context of designing a clinically compatible GI‐based BCT system, the metric is applicable beyond this specific modality and can find use in various imaging tasks. Using this framework, a comprehensive grid search is performed to determine the optimal system parameters. The remainder of this paper is structured as follows: First, a brief introduction to GI and its current challenges is provided. Next, the proposed metric is detailed, followed by its application to the optimization of a new clinical system. Finally, the identified configuration is benchmarked against a commercial BCT scanner and a previously published GI system in an in silico study using an anthropomorphic breast phantom.

## 2 GRATING INTERFEROMETRY

**FIGURE 1:**
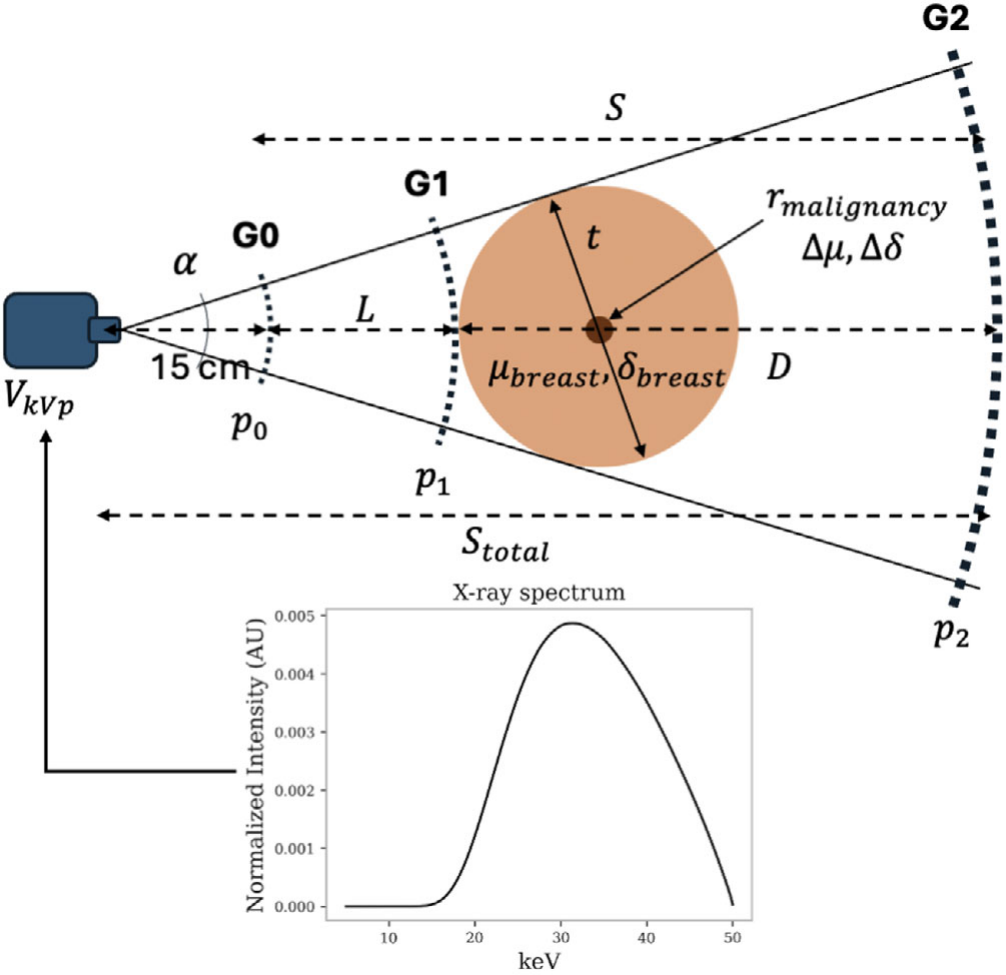
Schematic of a GI system and all the parameters used in the grid search analysis. By iterating over $p_{0}$, L, λ, and $T_{O}$, all remaining parameters are determined. Using these values and the defined acceleration voltage for a tungsten source with a 3 mm Al filter, the CNRD is then computed as described in Equation (3). CNRD, contrast‐to‐noise ratio weighted by dose; GI, grating interferometry.

Grating interferometry in the Talbot–Lau configuration^9^ employs three gratings: a source grating (G0) to enhance spatial coherence, a phase grating (G1) to modulate the wavefront and generate a periodic interference pattern (the Talbot carpet), and an analyzer grating (G2) typically in front of the detector to sample this pattern. By laterally translating one of the gratings in discrete steps, a phase‐stepping curve (PSC) is generated, enabling the extraction of phase‐contrast information from the recorded intensity variations. A schematic representation of this setup is provided in Figure 1.

The sensitivity of a GI is directly linked to the pitch of G2 and the distance of the sample to it,^21^ which can introduce design trade‐offs. Long devices in clinical settings are impractical and consequently, gratings with smaller pitches are required. At the same time, to block x‐rays effectively, especially at higher energies, absorption gratings must have sufficient height, with the absorbing material usually being gold. The fabrication of high‐aspect‐ratio (HAR) gratings is technically challenging and poses significant manufacturing difficulties. Specifically, producing HAR gratings can lead to severe artifacts in the grating geometry, which ultimately affect the image quality in GI.^20^ As a result, the challenging microfabrication of arbitrarily HAR gratings imposes design constraints on GI systems.

## 3 METHODS

In this study, the design and optimization of a computed tomography system for breast imaging based on grating interferometry is presented. The system is developed using a unified optimization framework, which simultaneously leverages both absorption and phase‐contrast imaging channels to enhance performance.

The absorption signal recorded at the detector plane follows the Beer–Lambert law. For a sphere with radius r and material M, the total integrated signal over the complete detector area while considering infinitesimally small pixels is

(1) $$S_{T}=\frac{4}{3}\pi r^{3}\mu ,$$

which corresponds to the volume of the sphere weighted by the linear attenuation coefficient μ. Similarly, integrating the complete differential phase contrast signal yields the total refraction signal:

(2) $$S_{\varphi }=4\pi r^{2}\frac{d}{p}\delta ,$$

where δ is the refractive index decrement from the complex refractive index n=1−δ+iβ. Notably, in contrast to the attenuation signal, the phase contrast signal depends on the surface area of the sphere, weighted by the system sensitivity and the refractive index decrement. The complete derivation can be found in the supplementary material.

When distinguishing two materials, their differences in linear attenuation coefficient and refractive index decrement are typically calculated. Considering noise, this comparison leads to the contrast‐to‐noise ratio (CNR). In clinical imaging, dose is a crucial factor in determining the feasibility of an investigation. Following the ALARA principle—keeping radiation exposure ”as low as reasonably achievable”—it is essential to optimize image quality while minimizing dose. Consequently, the CNR is often weighted by dose, resulting in the contrast‐to‐noise ratio weighted by dose (CNRD). To maximize the contribution of both contrast modalities, the following optimization metric is introduced:

(3) $$CNRD=\left(\frac{S_{T_{\Delta \mu }}}{\sigma_{T}}+\frac{S_{\varphi_{\Delta \delta }}}{\sigma_{\varphi }}\right)\sqrt{\frac{1}{Dose}},$$

where Δμ and Δδ represent the contrast differences between two materials and replace μ and δ in Equations (1) and (2), respectively. The noise terms, or uncertainty terms, are defined as in ref. [22]:

(4) $$\sigma_{T}=\frac{1}{\sqrt{I_{D}}},$$

(5) $$\sigma_{\varphi }=\frac{1}{\sqrt{I_{D}}V_{eff}},$$

where $V_{eff}$ is the effective visibility after the sample under a polychromatic spectrum, and $I_{D}$ is the number of photons reaching the detector. The visibility of an interferometer depends on both the quality of the gratings and the energy spectrum. The number of detected photons and the absorbed dose are influenced by sample thickness and material composition. A previous study investigated the optimal CNR as a function of tube acceleration voltage and sample thickness in BCT.^23^ Building on this, the present work focuses on optimizing key system parameters—including tube voltage, design energy, grating pitch, and system geometry—across a range of breast sizes. The primary objective is to maximize the CNRD between fibroglandular and tumor tissues, as differentiating between these tissue types remains a significant diagnostic challenge. Dose considerations are incorporated to assess whether grating interferometry enhances contrast while accounting for the performance trade‐offs introduced by the analyzer grating.

### 3.1 Grid search

A grid search is a brute‐force optimization technique used to explore a defined parameter space by exhaustively evaluating all possible combinations of specified parameter values. It involves systematically varying each parameter across a set of discrete values and assessing the performance of the system or model at each combination. This approach ensures that the best‐performing configuration within the predefined grid is identified, although it can be computationally intensive, especially when dealing with high‐dimensional parameter spaces. In this work, the optimization metric defined in Equation (3) is applied to the parameter space defined in Table 1. To reduce computation time, various approximation methods are implemented for estimating the different variables in the metric.

**TABLE 1:** Parameter space and constraints used for the grid search.

Parameter	Value	Constraint
Pitch $p_{0}$	3–10 μm	Height requirement to meet $\frac{3}{\mu_{\text{Au}}\left(E_{d}\right)}$ with AR 50
Distance G0–G1 L	10–100 cm	Total system length 1.45 m
Design energy $E_{d}$	20–46 keV	Grating manufacturing AR 50
Talbot order $T_{O}$	1, 3, 5, or 7	Total system length of 1.45 m
Distance source ‐ G0	15 cm	Limited by bending of silicon wafers
Field‐of‐view FOV	20 cm	Requirement to fit large samples
Acceleration voltage $V_{kVp}$	25 – 70 kVp	

*Note*: Except for the acceleration voltage, all other parameters of the interferometry system influence each other.

To ensure feasibility, constraints are placed on the maximum system length and grating parameters, preventing the selection of designs that cannot be manufactured. As indicated by Equation (2), the PC signal increases with a larger sample‐to‐G2 distance and a smaller grating pitch. However, without constraints, this could lead to impractically long systems and extremely small pitches. To maintain clinical feasibility, the system length is limited to 1.45 m, while the grating design is constrained to a maximum aspect ratio of 50 (i.e., grating height over half the pitch cannot exceed 50).

The system was optimized for an inverse geometry configuration,^24^ with the sample placed after G1 to ensure that additional photon absorption occurs only at G2, thereby reducing the dose penalty introduced by the gratings. Additionally, this geometry places the smallest pitch $p_{0}$ at the G0 level, where the required active area is minimized compared to the G2 grating. The remaining interferometer parameters can be determined from $p_{0}$, the distance L between G0 and G1, the Talbot order $T_{O}$, and the design energy $E_{d}$ as follows:

(6) $$S=\frac{L\cdot T_{O}\cdot p_{0}^{2}}{T_{O}\cdot p_{0}^{2}-2\lambda \cdot L},$$

(7) $$p_{1}=\frac{2\cdot D\cdot p_{0}}{S},$$

(8) $$p_{2}=\frac{p_{0}\cdot D}{L},$$

(9) D=S−L,

where λ is the wavelength corresponding to $E_{d}$, S is the interferometer length, D is the distance between G1 and G2, and $p_{1}$ and $p_{2}$ are the grating pitches of G1 and G2, respectively. First, all $E_{d}$ values are iterated to determine the minimum required grating height, defined as $\frac{3}{\mu_{\text{Au}}\left(E_{d}\right)}$, which ensures at least 95 % photon absorption and prevents the visibility of the interference pattern from being penalized by insufficient height. This minimum height per design energy constrains the allowable pitches for G0, as the grating height must remain within the limit imposed by an aspect ratio (AR) of 50. If this constraint is satisfied, the interferometer parameters are computed according to Equations (6) – (9). Additionally, the total system length is verified to remain below the maximum allowable value, that is, $S_{\max}=S+0.15\;m$.

With the allowed parameters, Equation (3) is computed. A polychromatic spectrum is used, where the acceleration voltage is an iteration parameter. The spectrum arises from a tungsten target filtered with 3 mm of aluminum. The spectrum is generated using the *SpekPy* Python package.^25^ Using this spectrum, the effective linear attenuation coefficient and effective refractive index decrement for breast tissue and malignancies are determined by weighting them with the normalized spectrum and visibility distribution, as follows:

(10) $$\Delta \mu_{\text{eff}}=\int w\left(E\right)\Delta \mu \left(E\right)dE,$$

(11) $$\Delta \delta_{\text{eff}}=\frac{\int w(E)V(E)\Delta \delta (E)dE}{\int w(E)V(E)dE},$$

where w(E) is the normalized spectrum, and V(E) represents the visibility spectrum for a given $E_{d}$ and $T_{O}$. To obtain a realistic visibility distribution for a Talbot–Lau interferometer with gratings of a given AR, pitch, and duty cycle, wave‐propagation simulations need to be used, as demonstrated in previous works.^20^, ^26^ However, conducting a grid search under these conditions would require several hundred wave simulations, making the process computationally expensive, especially for a polychromatic spectrum. As a first approximation, the gratings are modeled as rectangular shaped with a duty cycle of 50 %, and the analytically derived upper limit from Thuering et al.^27^ is used:

(12) $$V\left(E\right)=\frac{2}{\pi }\left|\sin{\left(\frac{\pi }{2}\frac{E_{d}}{E}\right)}^{2}\sin\left(\frac{\pi T_{O}}{2}\frac{E_{d}}{E}\right)\right|.$$

The linear attenuation coefficients for breast tissue are obtained from the ICRU‐44 database.^28^ The linear attenuation coefficient and refractive index decrement for adipose, fibroglandular, and tumor tissue are taken from Johns et al.^29^ and Willner et al.,^30^ respectively. Willner et al. introduced the concept of Hounsfield units for phase contrast $HU_{p}$, which relate the electron density of water to that of the tissue.^30^ Since the refractive index decrement is directly linked to electron density and energy, it can be determined as follows:

(13) $$\delta \left(E\right)=\frac{r_{0}h^{2}c^{2}}{2\pi E}\rho_{e},$$

where $r_{0}$ is the classical electron radius, h is Planck's constant, c is the speed of light, and $\rho_{e}$ represents the electron density of the material, which is derived from $HU_{p}$ as:

(14) $$\rho_{e}=\left(1+\frac{HU_{p}}{1000}\right)\rho_{e,\text{water}}.$$

As the spectrum is hardened when propagating through the breast, the effective attenuation coefficient and refractive index decrement will change. To account for the attenuation caused by the breast, the normalized effective spectrum is used in Equations (10) and Equation (11), defined as follows:

(15) $$w_{eff}\left(E\right)=\frac{w\left(E\right)\exp(-\mu_{\text{breast}}\left(E\right)t)}{\int w\left(E\right)\exp(-\mu_{\text{breast}}\left(E\right)t)dE}.$$

Note, in the case of GI, hardening from the gratings is also considered. Using the two spectra, the visibility of the system with and without the sample can be determined as follows:

(16) $$V_{\text{eff}}=Q\int V\left(E\right)w_{N}\left(E\right){\left(1-\exp(-\mu_{\text{Au}}h_{g})\right)}^{2}dE,$$

where $w_{N}\left(E\right)$ corresponds to either the normalized hardened spectrum $w_{eff}$ or the normalized original spectrum w. To compensate for the finite height $h_{g}$ of the gratings, which cannot absorb the highest energy photons, a penalty is applied to the visibility spectrum. The squared term arises from the use of two absorption gratings (G0 and G2). The visibility spectrum derived from Equation (12) represents an optimistic estimate, as it assumes ideal grating absorption—that is, that the absorbing regions completely block all photons. Moreover, the individual visibility values across the energy range are consistently higher than those demonstrated to be feasible in simulations, even after correcting for the grating transmission (see Figure S1). To account for this, the total effective visibility (Equation 16) is scaled by a quality factor Q, introduced to align with the simulated visibility. This factor also incorporates a penalty reflecting grating imperfections, ensuring that the effective visibility remains within experimentally observed ranges.^20^, ^26^ In this study, Q is empirically set to 0.65. Further experimental investigations are planned to refine the Q factor and its physical meaning.

Finally, the remaining two quantities to be determined are the absorbed dose and the intensity at the detector $I_{D}$. Both are influenced by the system geometry—specifically, the fan angle α for dose and the total system length for intensity. In this model, the breast is approximated as a cylinder and the malignancy as a sphere. However, only the cylindrical breast volume is considered for dose and intensity calculations. The fan angle α is defined as twice the angle formed between the lines extending from the source to the sample center and the line passing tangentially to the sample, as illustrated in Figure 1. Additionally, a fixed sample height is assumed in this work, leading to an azimuthal opening angle of β. Using these angles and the total system length $S_{total}$, the dose and intensity at the detector are determined as follows:

(17) $$Dose=\alpha \beta \int w\left(E\right)(1-\exp\left(-\mu_{\text{breast}}\left(E\right)t\right))EdE,$$

(18) $$I_{D}=\frac{1}{S_{{\text{total}}^{2}}}\int w\left(E\right)\exp\left(-\mu_{\text{breast}}\left(E\right)t\right)dE.$$

**FIGURE 2:**
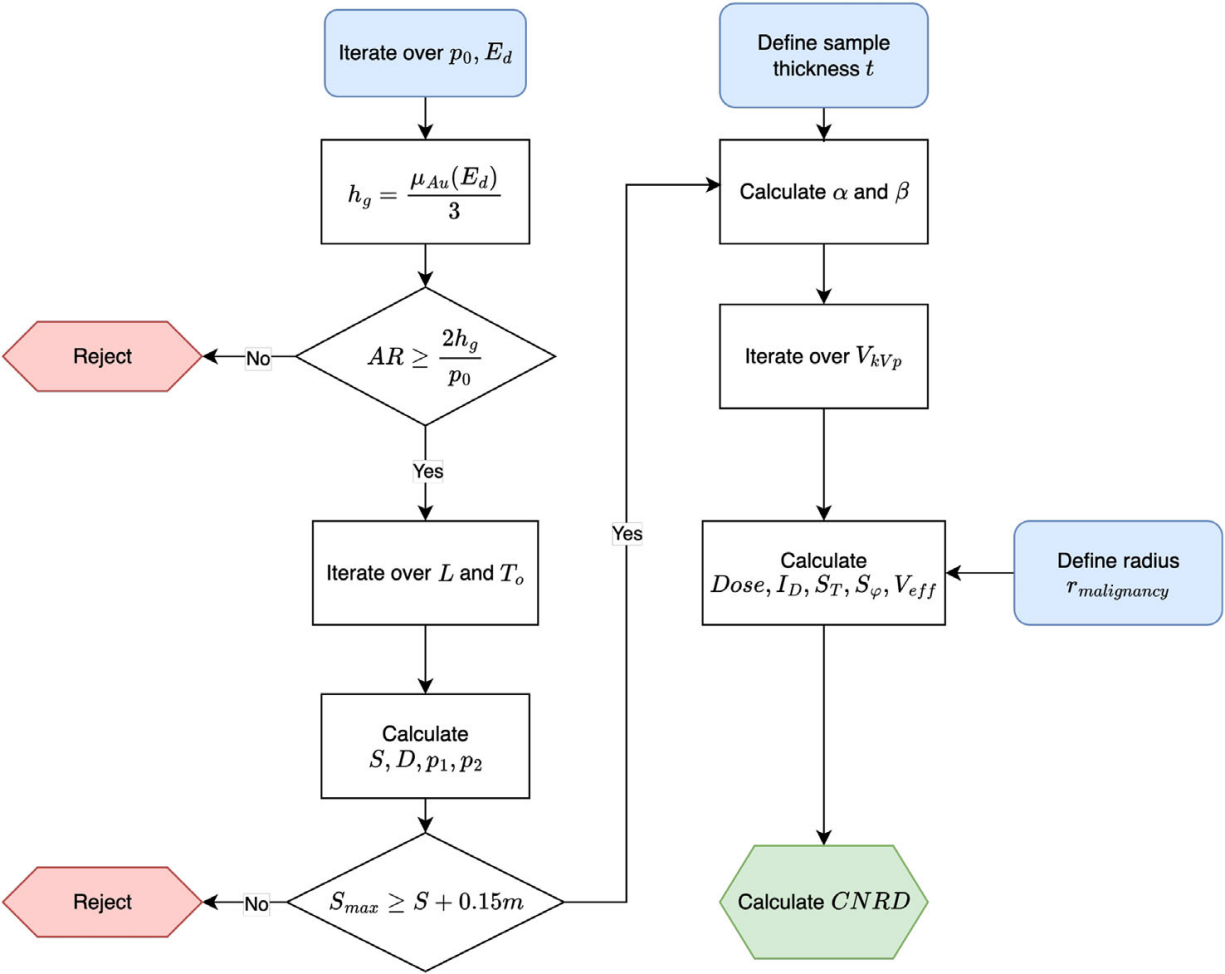
Flowchart illustrating the grid search process. The initial parameters are the pitch of G0 and the design energy. Based on these values, the grating height is determined and assessed against manufacturing constraints. Subsequently, the system lengths and the pitches of G1 and G2 are computed, ensuring that the maximum system length is not exceeded. The opening angle is then determined according to the sample thickness and system geometry. Finally, an iteration over all acceleration voltages is performed, computing the CNRD values, where $S_{\mu }$ and $S_{\varphi }$ depend on $r_{malignancy}$. CNRD, contrast‐to‐noise ratio weighted by dose.

For the dose calculations, spectral hardening is introduced by the G0 and G1 gratings, while for $I_{M}$, the beam is further hardened by G2. In Equations (17) and (18), only the central part of the sample is considered for calculating transmitted intensity and absorbed dose. The specimen mass is not included in the calculations, as it remains the same across all systems where the breast diameter t is constant. With all parameters defined, Equation (3) can be computed. The complete process is illustrated in Figure 2.

### 3.2 In silico reconstruction simulations

**FIGURE 3:**
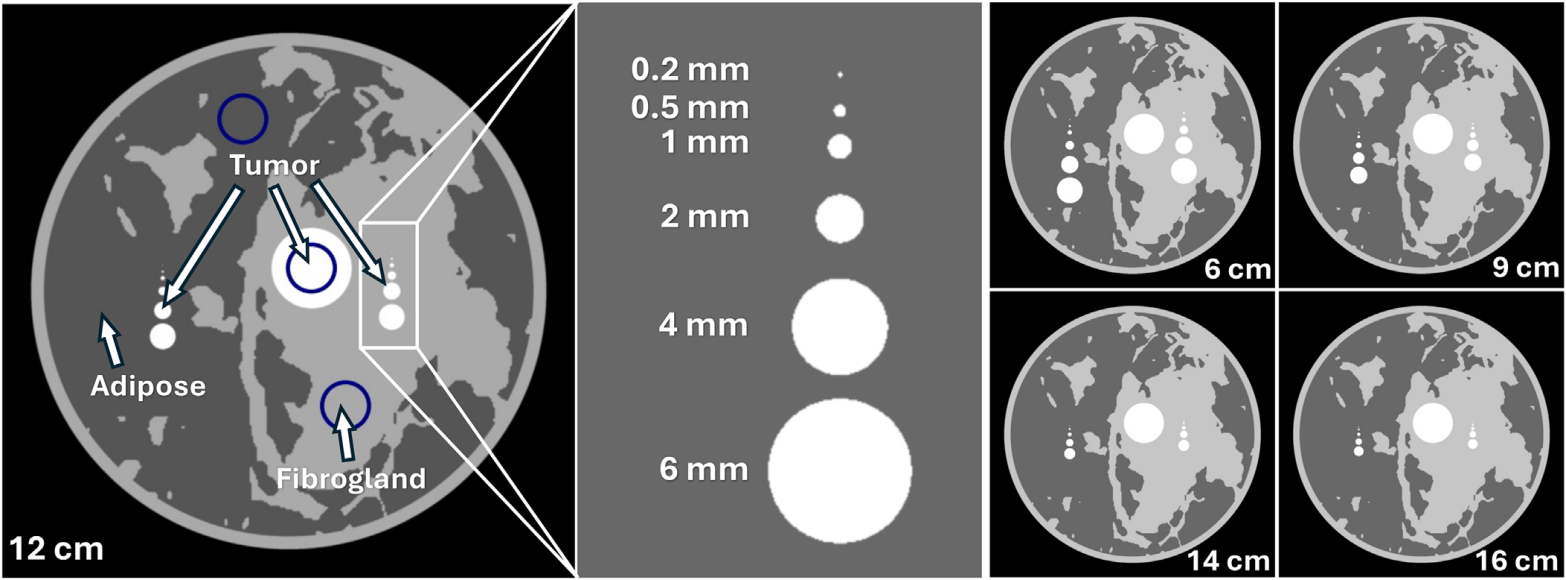
Phantom used to simulate reconstructions with various doses on the different systems. The phantom consists of three materials; tumor tissue, fibroglandular tissue, and adipose tissue. The structure from the fibroglandular tissue was created using the same approach as in ref. [54]. Next to a large region of tumor tissue, smaller areas were added to simulate small malignancies embedded in adipose and fibroglandular tissue. When simulating different breast diameters, the complete sample is scaled to the corresponding size, while the small tumor inserts stay the same. The blue circles indicate the regions where the CNR was calculated. CNR, contrast‐to‐noise ratio.

The CNRD provides a quantitative metric based on analytical models and approximations. To assess image quality, simulations are performed on a phantom composed of breast‐like materials with varying feature sizes, allowing evaluation of resolution as a function of the applied dose, similar to ref. [19]. The phantom is depicted in Figure 3.

The simulation involves forward projecting the phantom and reconstructing it using filtered back projection with the Ram–Lak^31^ and Hilbert filter^32^ for absorption and refraction, respectively. To account for the polychromatic nature of the spectrum, different approaches are applied to the two contrast channels. For each energy, a sinogram is generated based on the corresponding δ and μ values by forward projecting the phantom. The energy bin is set to 1 keV. For attenuation, the projected line integral at each angle, position, and energy is computed as:

(19) $$P_{\mu_{E,x,y,\varphi }}=\exp\left(-\int \mu (E,x,y,z)dz\right).$$

Eventually, all sinograms are weighted by the spectrum and then summed to obtain the polychromatic sinogram of attenuation.

For refraction, the initial spectrum is hardened at each detector pixel and projection angle by replacing the exponential term in Equation (15) by Equation (19). Next, the normalized hardened spectrum is weighted by the initial visibility spectrum without the sample. Since the analytical visibility spectrum in Equation (12) provides an upper limit for all energies, a more realistic visibility spectrum for different systems is simulated using the *RaveSim* package.^26^ The hardened spectrum is then weighted by the visibility spectrum and normalized so that the cumulative sum equals to 1 leading to $w_{V_{x,y,\varphi }}\left(E\right)$. The individual projections for different energies are differentiated and weighted by $w_{V_{x,y,\varphi }}$ to obtain a polychromatic differential phase contrast sinogram as^33^:

(20) $$\begin{array}{cc} & P_{\delta_{x,y,\varphi }}=\\ & \arg\left(\int w_{V_{x,y,\varphi }}\left(E\right)\exp\left(i\frac{2\pi d}{p_{2}}\frac{\partial }{\partial x}\int \delta \left(E,x,y,z\right)dz\right)dE\right), \end{array}$$

where d represents the distance between the sample and G2, $p_{2}$ denotes the pitch of G2, and i is the imaginary unit. The sensitivity scales linearly with distance, so the differential phase contrast signal of a lesion changes with the distance to G1 (or G2). However, in a CT scan, as simulated here, it is correct to define the distance from the centre of rotation to G2 for all points on the sample. Over a full 360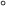 scan, each point in the sample is, on average, equidistant from G1, accounting for the fact that the sensitivity scales linearly with distance.^24^

As reported in ref. [34], absorption CT images exhibit a better modulation transfer function (MTF) compared to reconstructed refraction images when using a Talbot–Lau system with a π‐shifting G1 grating. To account for this difference, the refraction signal in Equation (20) is blurred using a Gaussian filter with a kernel size determined by matching the resolution from absorption to the one from refraction at 10 % MTF. For this, the *RaveSim* package is used and the results can be found in the Supporting Information. Additionally, to include the visibility reduction effect arising from the second derivative of the phase signal, Equation (20) is additionally differentiated in x to yield the reduction $D_{\delta^{\prime \prime }}$.^35^

Finally, the phase stepping curve of the system is modeled according to:

(21) $$I_{x,y,\varphi ,k}=\frac{P_{\mu_{x,y,\varphi }}}{N}\left(1+V_{x,y,\varphi }D_{\delta^{\prime \prime }}\cos\left(P_{\delta_{x,y,\varphi }}+\frac{2\pi k}{N}\right)\right)$$

where k corresponds to the current phase step, N is the total number of phase steps, and $V_{x,y,\varphi }$ is the effective visibility after the sample for each pixel and projection. This equation simulates the mechanical stepping of the grating over one period. For each phase step and pixel, Poisson‐distributed noise is incorporated into the data based on the number of photons detected per pixel. From the noisy phase‐stepping curves, the attenuation and differential phase contrast sinograms are extracted and subsequently reconstructed using filtered back projection with the ASTRA toolbox^36^ in cone‐beam geometry.

### 3.3 Image fusion and dose evaluation

The in‐plane noise characteristics of attenuation and phase contrast differ due to the intrinsic nature of the differential signal.^37^ The noise power spectrum (NPS) for phase contrast is more pronounced at lower frequencies, whereas attenuation exhibits most of its noise at higher frequencies. This difference motivates the fusion of both contrast channels, as demonstrated in ref. [19], and is briefly explained here. The fused image is obtained by summing a high‐pass filtered phase contrast image with a low‐pass filtered attenuation image, both processed using Gaussian kernels of the same size. The kernel size is selected to minimize the noise power spectrum while preserving structural details. To account for differences in gray values, both channels are normalized so that the tissues of interest are scaled between 0 and 1, where the lower‐valued tissue is set to 0 and the higher‐valued tissue to 1. A more detailed explanation can be found in the Chapter S6.

All X‐ray spectra were generated using *SpekPy* ^25^ considering a 3 mm Al filter. Based on these spectra, the total photon counts were calculated and matched to the dose measurements reported in.^19^ Using these measurements, the doses for the different setups were calculated following the approach described in the Supporting Information.

An important aspect not addressed in the grid search is the number of photons reaching the detector at very low doses, as the normalization by the dose yields a measure of the system's dose efficiency that is independent of the absolute number of incident photons. The differential phase contrast signal φ is periodic and uniquely defined within the interval ]−π,π]. The retrieved signal may fluctuate in this range at high noise levels (i.e., low radiation dose or high attenuation), invalidating the noise dependence in Equation (5). In the limit $I_{M}\to \;0$, the retrieved signal follows a uniform distribution with $\sigma_{\varphi_{\text{Max}}}=\pi /\sqrt{3}$, resulting in a complete loss of phase information.^37^ This phenomenon differs for attenuation, as there is no upper limit on $\sigma_{T}$.

To ensure sufficient statistical accuracy at low doses, even for large breast diameters, an empirical threshold of $\sigma_{\varphi_{\text{Max}}}/2.5$ was set to guarantee reliable information retrieval as experimental observations showed that signals could still be extracted at these uncertainty levels.^19^ The mean number of photons required per phase step at a single detector pixel is influenced by the interferometer's visibility, with higher visibility enabling a reduction in the necessary photon count.

### 3.4 Image evaluation metric

The reconstructed images were quantitatively evaluated following the approach of Rawlik et al.^19^ and Raupach et al.,^38^ based on the Rose criterion: a CNR of 5 between two tissue types is required to resolve breast morphology.^39^ The analysis was conducted for three tissue type pairs: adipose versus fibroglandular, adipose versus tumor, and fibroglandular versus tumor. For all analyses, the images were scaled such that the tissue type with the higher value was set to 1, while the other was set to 0. This same scaling procedure was applied when fusing images to maintain a consistent dynamic range across contrast channels. Gaussian filtering was then applied to the individual images at each dose level to enhance the CNR to the targeted value, while ultimately noting the kernel size required. Larger kernels introduce a point spread function (PSF), limiting the imaging resolution. This relationship allows for a lower resolution limit derivation by determining the full‐width at half‐maximum (FWHM) of the Gaussian kernel required to achieve a CNR of 5 at each dose level and region (blue rings in Figure 3).

## 4 RESULTS

### 4.1 Grid search analysis

The grid search explored various parameter combinations within the constraints and ranges defined in Table 1 (optimization for longer systems is provided in the Figure S1). The iteration step sizes were set to 1 keV for the design energy, 0.1 μm for the $p_{0}$ pitch, and 500 μm for the G0–G1 distance L, resulting in 393 distinct combinations. Iterations over the acceleration voltage were performed with a step size of 1 kVp. As benchmarks, an early GI breast CT system by Rawlik et al.^19^ and the commercially available “nu:view” breast CT scanner (AB‐CT–Advanced Breast‐CT GmbH, Erlangen, Germany) with specifications from^40^ were used. For the latter, the absence of gratings was accounted for, which increases flux and eliminates the dose penalty from the G2 grating.

**FIGURE 4:**
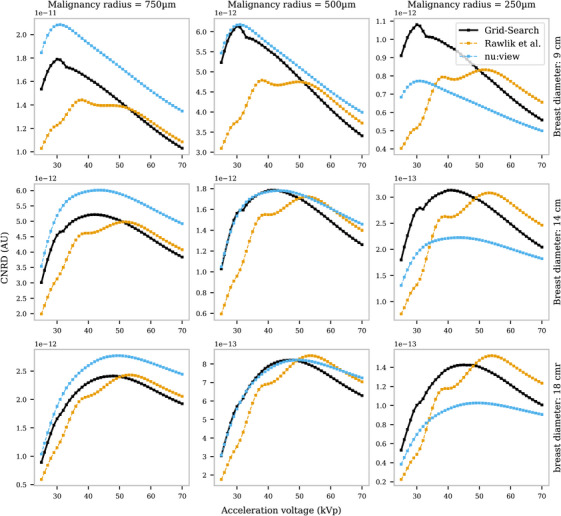
The CNRD values for different breast sizes and malignancies were evaluated for a system with a maximum length of 1.45 m. Each plot illustrates the calculated CNRD as a function of the acceleration voltage, comparing the two benchmark systems with the best‐performing systems identified at each acceleration voltage in the grid search. CNRD, contrast‐to‐noise ratio weighted by dose.

For all acceleration voltages, the best‐performing system was selected based on Equation (3), and the CNRD was plotted against acceleration voltage compared to the benchmark systems. Performance was analyzed for different breast diameters (9, 14, and 18 cm) and malignancy radii (0.75, 0.5, and 0.25 mm), with results presented in Figure 4. When comparing the impact of malignancy size, a larger radius resulted in increased efficiency of absorption‐based CT, surpassing GI breast CT across all acceleration voltages. However, the refraction signal for smaller malignancies contributed to a higher CNRD across all acceleration voltages than the CNRD of the commercial system. This demonstrates the potential of grating interferometry to enhance diagnostic performance in high‐resolution imaging tasks, especially when subtle tissue differences must be resolved. With increasing breast diameter, the optimal acceleration voltages increases as derived from the CNRD calculations. These results align with findings from polychromatic simulations on absorption‐based systems.^23^

### 4.2 Reconstructions and CNR‐analysis

A system optimized for clinical applications was selected based on the grid search results and is from here on referred to as GI‐BCT. To ensure that uncertainty remains below $\sigma_{\varphi_{\text{Max}}}/2.5$ at dose levels attractive for diagnostics, the minimum acceleration voltage was determined as outlined in the Supporting Information, which resulted in a minimum acceleration voltage of 50 kVp. The new system, along with benchmark systems and their setup parameters, is summarized in Table 2.

**TABLE 2:** Parameters of the systems that are compared in a simulation study with reconstructed images over various dose levels.

	GI‐BCT	Optica‐2023	AB‐CT
Source–sample‐distance	89.6 cm	101.8 cm	50 cm
Sample–detector‐distance	54.6 cm	71.8 cm	15 cm
Acceleration voltage	50 kVp	70 kVp	60 kVp
Filtering	Al 3 mm	Al 3 mm	Al 3 mm
Pixel size	200 μm	200 μm	200 μm
Number of projections	1000	1000	1000
Grating pitches	5.3 μm	4.2 μm	None
Talbot‐order	3	5	None
Design energy	38 keV	46 keV	None
Effective visibility	23 %	10 %	None

Abbreviations: AB‐CT, advanced breast‐computed tomography; GI‐BCT, GI, grating interferometry‐breast computed tomography

**FIGURE 5:**
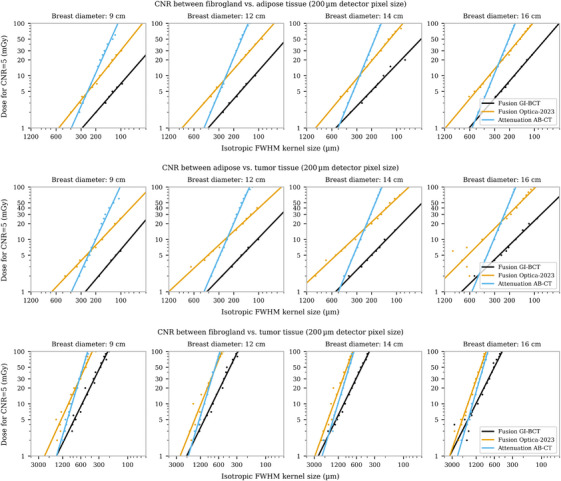
Dose versus isotropic kernel size required to achieve a CNR of 5, compared across different systems and various breast diameters. The dots represent evaluated kernel sizes through simulations, while the line corresponds to a first‐degree polynomial fit to these data points. CNR, contrast‐to‐noise ratio.

Using these parameters, reconstructed volumes of a simple phantom (Figure 3) were generated. Four different sample sizes were evaluated, with doses ranging from 5 to 100 mGy for each system. For the systems equipped with an interferometer (Optica‐2023 and GI‐BCT), the attenuation and refraction channels were fused to improve the NPS as explained in Chapter S5. From those images, the kernel size required to achieve a CNR of 5 was determined for each dose, as described in the methods section. The results are presented in Figure 5. For all simulations an MTF of 1 was assumed for the detector response, as no constraints apart from the detector pixel have been considered. Notably, the benchmarked systems utilize a 750 μm CdTe photon‐counting detector, whose quantum efficiency starts to decay after a energy of 50 keV,^41^ therefore mostly penalizing the benchmarked systems.

A first‐degree polynomial was fitted to the determined kernel sizes to derive the individual dose‐kernel curves. The slopes observed in the fused images from the GI systems differ from those of conventional attenuation‐based images, reflecting the distinct noise behavior as radiation dose increases. Additionally, as breast diameter increases, the intersection point between attenuation and fused image performance—previously reported by Rawlik et al.^19^—shifts toward higher dose levels. This trend suggests that while attenuation‐based imaging becomes increasingly advantageous for larger breast sizes, GI‐based fusion retains its strength in lower‐dose, high‐resolution scenarios.

**FIGURE 6:**
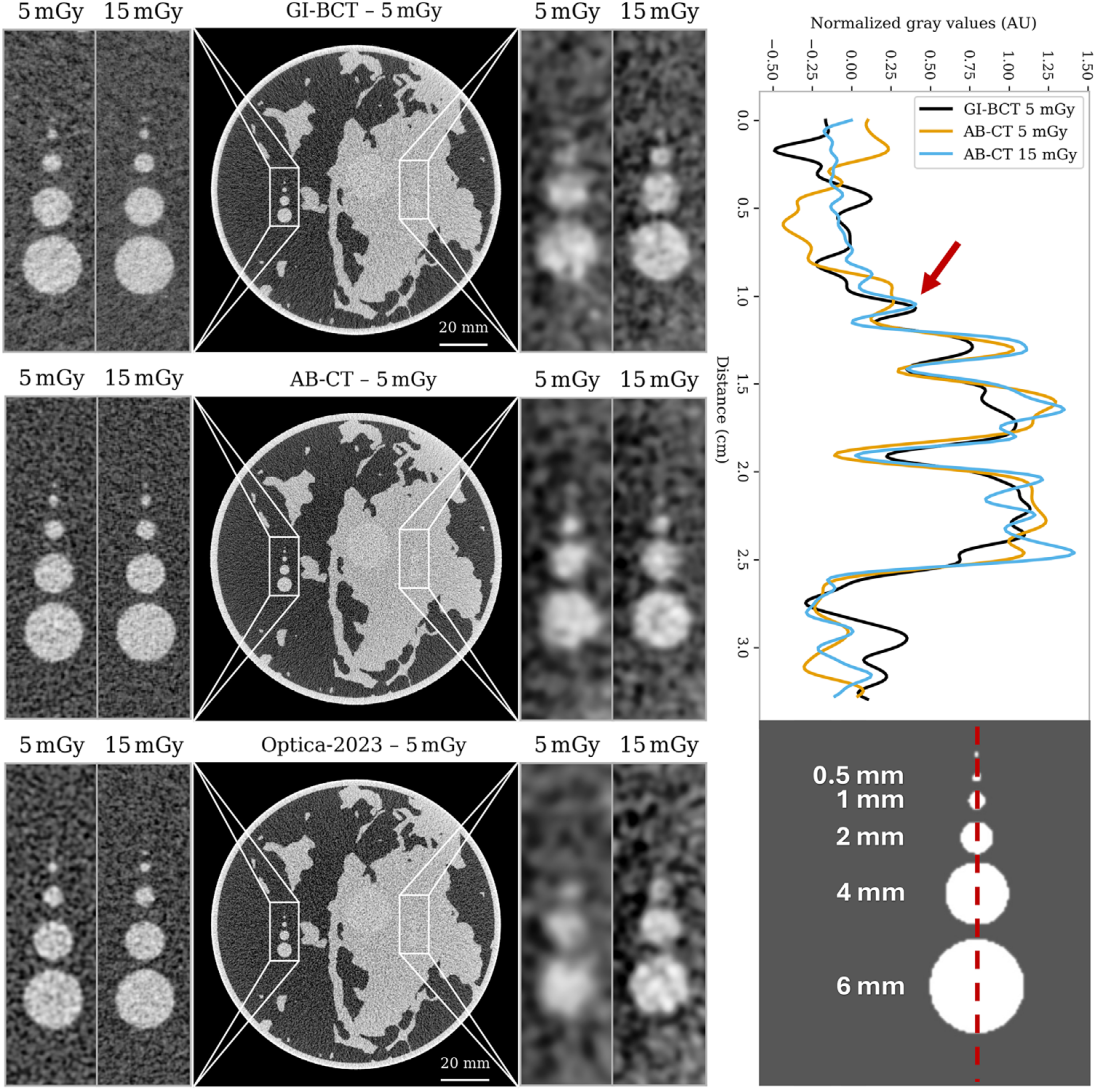
Simulated images of a 12 cm breast containing a tumor within both adipose and fibroglandular tissue. The left two panels depict tumors in adipose tissue, filtered using kernel sizes optimized for achieving a CNR of 5 between these tissues, as indicated in Figure 5. The entire slice is processed with kernel sizes tailored for differentiating fibroglandular and adipose tissue, while the right two zoomed‐in panels are filtered specifically for fibroglandular and tumor tissue. Each image is normalized such that the corresponding tissue types, as processed, have values between 0 and 1, with a consistent dynamic range applied across all systems. The top right plot shows line profiles through the tumor circles embedded in the fibroglandular tissue. Although the 1 mm tumor is clearly visible in the GI‐BCT image at 5 mGy, the AB‐CT system requires higher doses to achieve comparable detectability. AB‐CT, advanced breast‐computed tomography; CNR, contrast‐to‐noise ratio; GI‐BCT, GI, grating interferometry‐breast computed tomography.

Figure 6 depicts the image quality for a breast with a diameter of 12 cm, which closely aligns with the average effective breast diameter reported in Huang et al.^42^ The reconstructions were filtered using a Gaussian kernel, with kernel sizes determined from Figure 5 for the corresponding dose. The dots in Figure 5 represent the kernels required to achieve a CNR of 5 in the blue region defined in Figure 3. However, due to the stochastic nature of noise, outliers at specific dose levels can introduce excessive blurring. The kernel size was therefore defined from the fitted curve for each dose level to mitigate this effect.

The newly designed system demonstrates visually superior image quality compared to Optica‐2023. The high‐frequency details from the refraction signal yield sharper, more defined edges, especially at higher doses, compared to the AB‐CT system. These visual improvements are most apparent in smaller feature sizes as can be seen in the line profiles in Figure 6.

## 5 DISCUSSION

This work introduced an optimization metric to simultaneously enhance signal strength from absorption and refraction rather than prioritizing one over the other. The metric is applied to the design of a clinically compatible GI‐based BCT scanner. Hard constraints on grating fabrication were incorporated, limiting the AR to a maximum of 50 to ensure that high‐quality gratings can be manufactured. Based on these considerations, an improved system was designed, demonstrating superior image quality in resolution and dose efficiency for a given CNR over conventional BCT.

The CNRD from refraction and attenuation highly depends on the malignancy size. The refraction signal primarily originates from the surface rather than the volume, which aligns with the fact that refraction occurs at the interface between two materials and is influenced by surface morphology. As the malignancy size increases, the surface‐to‐volume ratio decreases, leading to a more substantial contribution from attenuation. Consequently, CNRD improves in attenuation for larger malignancies, making purely attenuation‐based imaging more favorable. This effect has been previously investigated by Raupach et al.^38^, who demonstrated that the required CNR ratio (i.e., the ratio of CNR from refraction to attenuation) must increase for larger structures to maintain equal observer performance. The detectability of larger objects in absorption imaging can be improved by reducing spatial resolution (e.g., filtering) as the NPS decreases at lower frequencies. However, blurring the refraction signal does not provide a similar benefit since the NPS remains high at those frequencies.

When examining the dependence on breast diameter, a decrease in the acceleration voltage is observed to be more favorable for smaller diameters. Since the refraction signal strength is highly dependent on system geometry, increasing the system length or reducing the pitch would significantly enhance the signal, as shown in the CNRD plot for a 2 m system in the Supporting Information (Figure S2). This configuration provides a higher signal for larger malignancies and improved performance for larger breast diameters. Yet, increasing the system length may not be a viable option for clinical use. With advancements in grating fabrication, further improvements in image quality compared to attenuation‐based imaging remain possible, as higher aspect ratios can be targeted, providing higher sensitivity for equal dose. Current grating techniques already enable comparable image quality, if not superior, to purely attenuation‐based imaging at the same dose.^19^

From the CNRD grid search, an optimal system is selected, and in the in silico reconstructions, the images are fused. A discrepancy exists in their relationship, as the CNRD metric considers the sum of the signals, whereas the image fusion selects the most suitable frequencies from each channel to reduce the NPS. Nevertheless, optimizing for CNRD also leads to improved fusion image quality. In the current fusion strategy, high frequencies are predominantly weighted by the refraction signal, which is also where the strongest signal contribution arises, especially for smaller malignancy sizes, as the CNRD increases primarily due to refraction. Equivalently, lower frequencies are more influenced by the absorption signal in the CNRD, which exhibits a lower NPS in that range. Therefore, maximizing the signal via CNRD inherently improves the overall quality of the fused image.

The optimized system demonstrates superior image quality across all dose levels, with performance approaching that of AB‐CT only at the lowest doses, as shown in Figure 5. As seen in the line profiles of Figure 6, achieving similar resolution at a CNR of 5 requires higher doses for AB‐CT due to the need for larger kernel sizes. This highlights the higher dose efficiency of the optimized system for equal resolution.

Compared to the Optica‐2023 system, the current design offers a significant improvement, despite being shorter and having larger grating periods. The system was designed to be more sensitive than a GI‐based breast CT system developed for a gantry by Büchner.^43^ That system was optimized using a metric adapted from Raupach et al.,^38^ which prioritized maximizing the CNR gain from refraction over attenuation. As a result, it favored designs operating at higher acceleration voltages, with a selected optimum at 70 kVp and a design energy of 46 keV. However, this approach introduces several challenges. As shown by Weigel et al.,^23^ the optimal acceleration voltage for absorption‐based breast CT in terms of dose efficiency (CNRD) lies between 30 and 40 kVp, substantially lower than the 70 kVp used in the earlier design. Additionally, higher photon energies reduce the refraction angle due to smaller refractive index decrements, thereby requiring higher sensitivity of the interferometer. To maintain this sensitivity, either the system length must be increased or the grating pitch reduced. The latter option additionally demands thicker gratings to maintain absorption efficiency at higher energies. These high aspect ratios are challenging to manufacture^44^, ^45^ and often introduce imperfections in the grating structures, ultimately degrading interferometer performance,^20^ particularly in the refraction signal. Reconstructed refraction and absorption images of the three systems are provided in the Chapter S8.

It is essential that sufficient photon statistics on the GI system are measured such that the uncertainty is below the asymptotic level of $\sigma_{\varphi_{\text{Max}}}$ (see Figure S5), with higher photon statistics needed for systems with low visibility. Insufficient photon counts can lead to cupping artifacts in the reconstructed images due to information loss in refraction (see Figure S8 for Optica‐2023 at 5 mGy). Consequently, fusing the signals becomes challenging, as the similarity in refractive index coefficients in conjunction with high noise amplitudes prevents proper normalization. Therefore, increasing photon statistics is necessary to leverage the signal arising from refraction. For low doses and large breast diameters, the only viable approach to reducing uncertainty caused by poor photon statistics is to increase the acceleration voltage. Nevertheless, the optimized system demonstrates superior CNR for breast diameters up to 16 cm, which encompasses the effective breast diameter of the vast majority of women. Higher diameters are primarily observed near the chest wall region.^42^ It can also be noted that for equal doses, the acceleration voltage for large breast diameters can be increased, with the caveat of reduced visibility but higher photon statistics. Similarly, clinical standards may increase the dose levels for larger samples as indicated by the European guidelines,^46^ thereby improving photon statistics per phase step and, with it, the refraction signal.

In this study, the influence of the dark‐field signal has not been considered, although it is an important parameter that affects the noise in refraction images by reducing the visibility. However, breast tissue is generally assumed not to generate any dark‐field signal, apart from beam hardening effects, which are accounted for in the simulations. In the context of breast imaging, microcalcifications are known to be a source of scattering.^16^, ^17^, ^18^ Modeling microcalcifications, however, is a non‐trivial task due to the lack of data on the extinction coefficient needed for in silico reconstructions, as performed in this work. Future investigations could explore the creation of a proper database, either through extensive wave‐propagation simulations or, more reliably, measurements conducted at synchrotron facilities.

The fusion strategy employed in this study was intentionally simple, assuming similar contrast behavior between attenuation and refraction channels. Although this assumption facilitates implementation, it overlooks the complex and often inverse contrast relationships observed between fibroglandular and tumor tissues, as previously reported by Willner et al.^30^ This inverse behavior introduces non‐linearities when combining histograms from both channels, complicating traditional fusion techniques, such as high‐ and low‐pass filtering. Moreover, the current method is based on a two‐material model that normalizes both contrast signals to a fixed range. This simplification fails in more heterogeneous tissue environments, where additional materials can lead to misrepresentations due to improper scaling of one or both channels. As a result, the fusion output may not accurately reflect the underlying tissue composition, particularly in complex diagnostic scenarios. To address these limitations, more advanced fusion techniques should be considered. One promising direction involves integrating fusion directly into the reconstruction process using a piecewise‐linear function within an intensity‐based iterative reconstruction framework^47^, ^48^ in conjunction with a plug‐and‐play denoiser^49^, ^50^ on the gradient of the iterations. This approach allows for a more quantitative and adaptive fusion process, improving image quality by enhancing noise suppression while preserving diagnostically relevant features. However, its pixel‐wise surjective nature limits its effectiveness in cases of inverse contrast behavior.

Alternatively, post‐reconstruction fusion methods offer greater flexibility and adaptability. Multimodal image fusion, which combines geometrically registered images from one or more modalities, provides a versatile framework for enhancing image quality. With the rapid advancement of deep learning, sophisticated fusion algorithms now offer the potential to more effectively exploit the complementary information in attenuation and refraction channels. These methods can produce high‐quality images that retain the full diagnostic value of both contrast mechanisms, ultimately improving lesion detectability and overall system performance.^51^

For the in silico investigation, the comparison metric is defined by the relationship between the applied dose and the isotropic kernel size to reach a CNR of 5. Although this approach provides a valuable initial quantitative assessment of overall image quality, it is important to acknowledge its limitations. Namely, it is not a task‐specific metric, as it ignores the size and shape of the lesion of interest and the noise texture. It primarily indicates how well the overall signal difference stands out above the noise. Therefore, future work should incorporate task‐based observer models, such as the channelized Hotelling observer,^52^ or directly evaluate image quality using the detectability index.^53^ These advanced metrics are better suited to predict human observer performance. Furthermore, with on‐going advancements in image reconstruction algorithms, particularly fusion algorithms with data‐driven priors, it would be highly beneficial to compare the resulting task‐based metrics against images obtained without grating interferometry.

## 6 CONCLUSION

This study demonstrates the potential of an optimized grating interferometry‐based breast computed tomography system to enhance image quality by effectively integrating attenuation and refraction contrast mechanisms. Through a systematic grid search and realistic simulation framework, the proposed design achieves superior contrast‐to‐noise performance, particularly for small malignancies, while maintaining dose levels appropriate for diagnostic imaging. The fusion of contrast channels further improves resolution and diagnostic clarity, outperforming conventional absorption‐based systems even at lower dose levels. These findings highlight the value of advanced optimization and fusion strategies in maximizing the diagnostic utility of grating interferometry and provide a foundation for extending this approach to other clinical imaging applications and beyond.

## CONFLICT OF INTEREST STATEMENT

The authors have no conflicts to disclose.

## Supporting information

Supporting Information

## Data Availability

The code of this study can be found on GitHub at https://github.com/eth‐xrm/gi_optimization.
