# Supplementary material for: Simultaneous signal optimization of refraction and attenuation in x‐ray grating interferometry: A case study for breast imaging
Source: Med Phys. 2025 Oct 9;52(10):e70069. doi: 10.1002/mp.70069 (PMC12509792; doi:10.1002/mp.70069)
Supplement: Supplementary file 1 — Supporting Information [file MP-52-0-s001.pdf]

Supplementary material: Simultaneous signal optimization of refraction and attenuation in X-ray grating interferometry: a case study for breast imaging

Alexandre Pereira[1,2], Michał Rawlik[1,2], Simon Spindler[1,2,3], Stefanie Kaser[1,2], Dominik Etter[1,2], Gianluca Iori[1,2], Martin Stauber[3], Lucia Romano[1,2], Marco Stampanoni[1,2]

[1]Institute for Biomedical Engineering, ETH Zürich and University of Zürich, Switzerland
[2]Swiss Light Source, Paul Scherrer Institute, Villigen, Switzerland
[3]GratXray, 5234 Villigen, Switzerland

Supplementary Material

1 Derivation signal attenuation and refraction

The amount of signal that can be obtained when imaging a sphere of radius r with both attenuation and refraction is evaluated. In the case of attenuation, the signal is defined as the reduction in intensity. For refraction, the signal is assumed to be proportional to the refraction angle, multiplied by the propagation distance d, and further modulated, as is typical in grating interferometry, by the inverse pitch of the analyser grating $1/p$.

The signal in attenuation is given as:

$$s = 1 - \frac{I}{I_0} = 1 - \exp(-\mu z), \tag{1}$$

where I and I_0 is the intensity recorded with and without the sample, respectively. The loss in intensity follows the Beer Lambert law, where μ is the linear attenuation coefficient of the sphere and z the distance it propagates through the sphere. For weak attenuation (i.e. $\mu z \ll 1$) the equation can be approximated to be $1 - \exp(-\mu z) \approx \mu z$. This is valid for soft-tissue and small objects. A projection of a sphere along the z direction (i.e. ray path direction) is given as:

$$P(x,y) = \begin{cases} \int_{-\sqrt{r^2-x^2-y^2}}^{\sqrt{r^2-x^2-y^2}} dz = 2\sqrt{r^2-x^2-y^2}, & \text{if } x^2+y^2 \leq r^2 \\ 0, & \text{otherwise.} \end{cases} \tag{2}$$

For a homogeneous sphere of a single material, the cumulative signal recorded on the whole detector area is given as:

$$S_T = \iint_{x^2+y^2\leq r^2} \mu P(x,y)\,dx\,dy \tag{3}$$

$$= \frac{4}{3}\pi r^3 \mu, \tag{4}$$

which results in the total signal of the sphere in attenuation to be equal to its volume multiplied by the linear attenuation coefficient.

The refraction angle α is the derivative of the projection of the imaginary part of the refractive index δ along the transverse direction x (i.e. perpendicular to the grating alignment):

$$\alpha(x,y) = \frac{d}{dx}\delta P(x,y) = \begin{cases} \frac{-2x}{\sqrt{r^2-x^2-y^2}}, & \text{if } x^2+y^2 \leq r^2 \\ 0, & \text{otherwise.} \end{cases} \tag{5}$$

The measurement of the refraction angle is based on changes in the detected signal intensity. Accordingly, a refraction-sensitive quantity is defined as the rate of change in intensity with

respect to the refraction angle for a generic refraction-sensitive system as:

$$\rho = \frac{ds}{d\alpha}. \tag{6}$$

Similarly to attenuation, the total cumulative signal from refraction is given as

$$S_\rho = \iint_{x^2+y^2 \le r^2} |\alpha(x,y)\rho| \, dx \, dy \tag{7}$$
$$= 2\pi r^2 \delta\rho, \tag{8}$$

where it can be noted, that the refractive signal is dependent on the surface of the sphere weighted by a factor $\frac{\delta\rho}{2}$. The absolute value ensures that no signal cancelation happens, as Eq. 5 is an odd function for x.

The signal for a grating interferometry (GI) system can be written as

$$s_{GI} = 1 - \left(\frac{V}{2} \sin\left(\frac{2\pi d\alpha}{p} \right) + \frac{1}{2} \right), \tag{9}$$

where V is the visibility of the interference pattern. The refractive sensitivity for a GI is then

$$\rho_{GI} = -\frac{d\pi V}{p} \cos\left(\frac{2d\pi\alpha}{p} \right). \tag{10}$$

The refraction sensitivity depends on the refraction angle, for which the average one is considered here

$$\bar{\rho_{GI}} = \frac{d}{p} \int_0^{\frac{p}{d}} \rho_{GI} \, d\alpha \tag{11}$$
$$= \frac{2dV}{p}. \tag{12}$$

Therefore, omitting the visibility, since it primarily affects the precision of the measurement (i.e., the noise characteristics) rather than the signal itself, the cumulative refraction signal in grating interferometry (GI) can be expressed as:

$$S_\varphi = 2\pi r^2 \delta \bar{\rho_{GI}} = 4\pi r^2 \frac{d}{p} \delta. \tag{13}$$

2 Visibility spectra

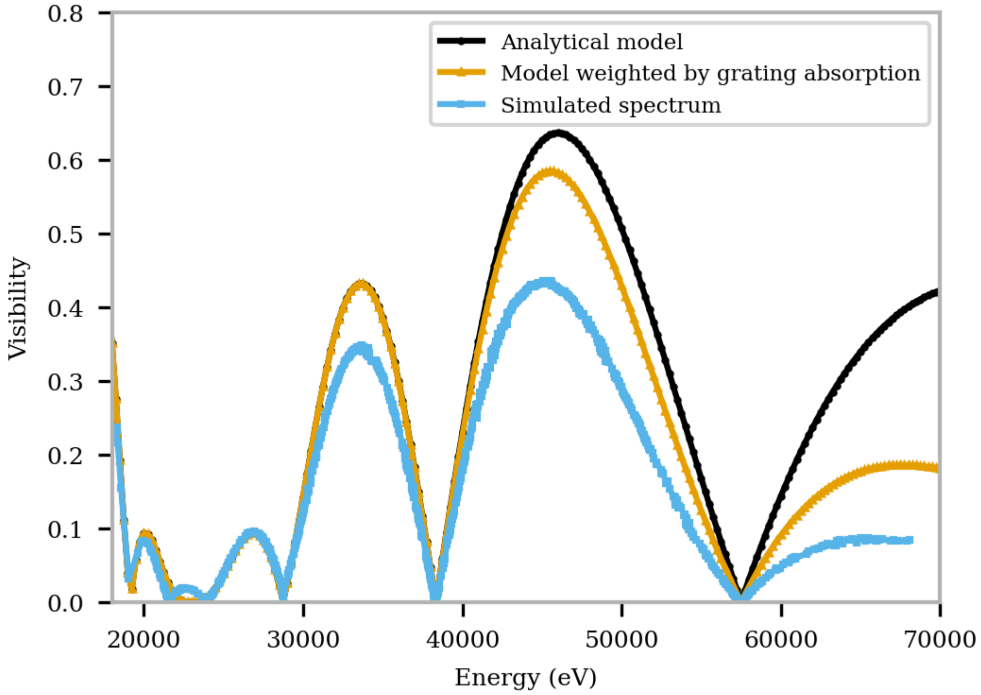

Figure S-1: The used visibility spectra in the grid-search follow an analytical model from [1]. However, this model provides an overestimation of the visibility per energy as it assumes a perfectly absorbing rectangular grating. Even by correcting for the grating absorption the values are higher than when calculating the visibility spectra with a wave-propagation simulation [2]. Therefore, for the grid-search an empirical quality factor is introduced to modulate the effective visibility to values observed in experiments.

3 CNRD for 2 m system

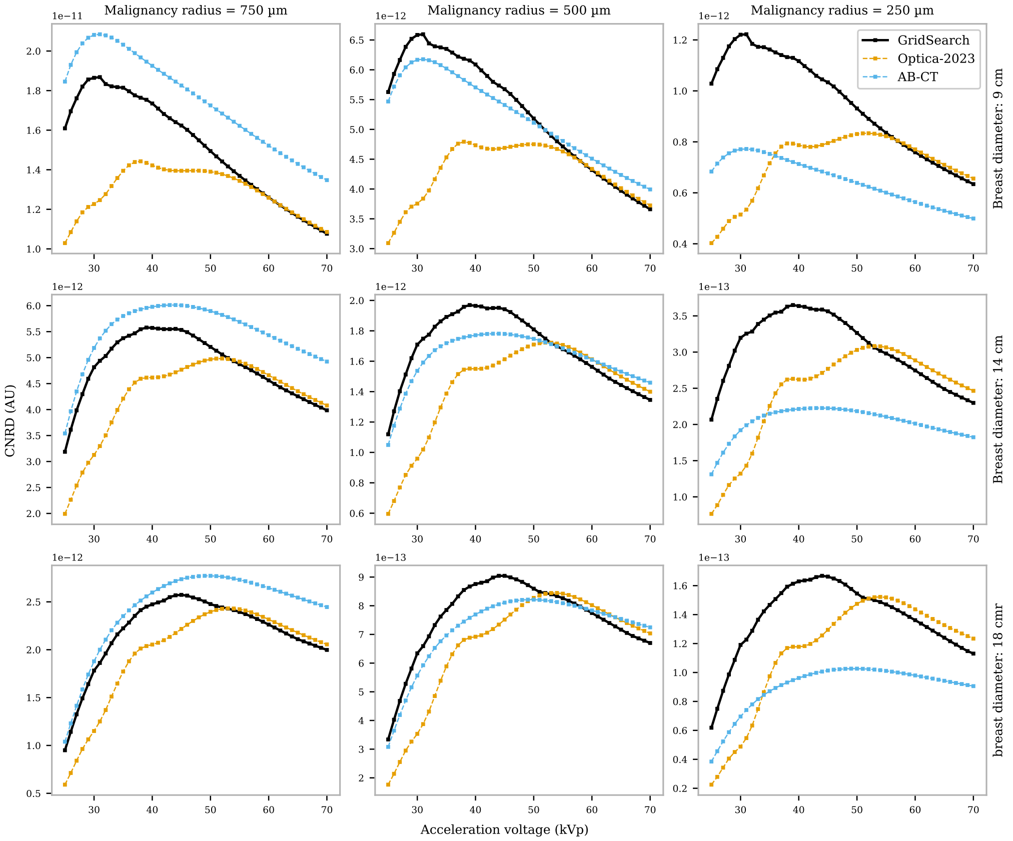

Figure S-2: The CNRD values for different breast sizes and malignancies were evaluated for a system with a maximum length of 2 m. Each plot illustrates the calculated CNRD as a function of the acceleration voltage, comparing the two benchmark systems with the best-performing systems identified at each acceleration voltage in the grid search. Different from the 1.45 m system is the increase in signal over all acceleration voltages, which comes from the fact that the signal increases with sample-G2-distance as defined in Eq. 5 from the main text.

4 Dose calculation

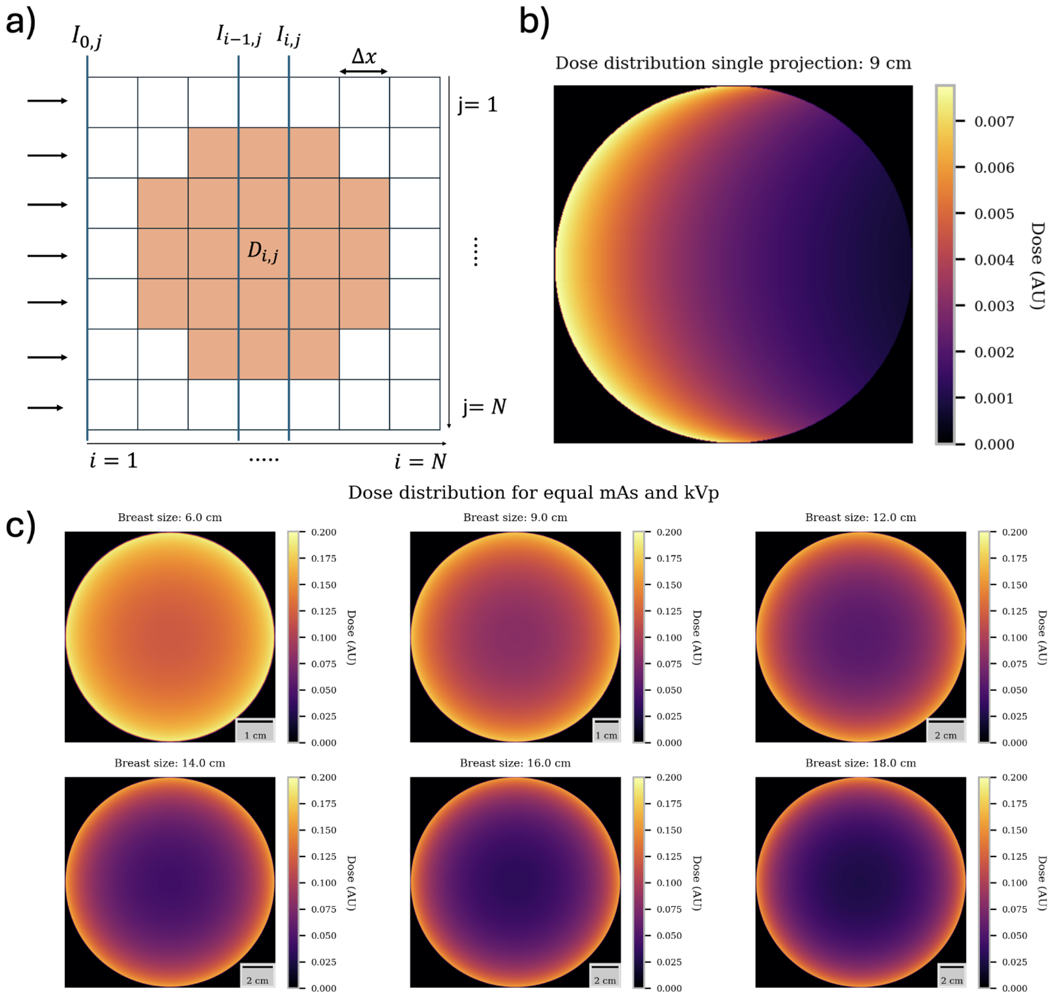

Figure S-3: a) Schematic representation of the dose calculation per projection and voxel. Each voxel is assigned a constant height equal to Δx. b) Dose distribution for a single projection angle. Since the object is rotationally symmetric, this distribution is transformed through rotation and summed to obtain the final dose distributions shown in (c). c) Dose distributions for equal mAs and 70 kVp across different breast diameters.

The dose in the simulations for different system geometries, acceleration voltages, and sample sizes is always referenced to the dose measurement from Rawlik et al. [3]. A simple rotationally symmetric phantom is considered for the dose calculation.

First, the fan angle α is determined, as illustrated in Fig. 1 of the main text. Using this, the total number of photons for each energy is extracted with the SpekPy package [5]. Next, a defined grid with spacing Δx and M projections is set up. The initial number of photons reaching a grid element (Fig. S-3a) is then calculated by dividing the total photon count by the number of grid columns and projections M. Finally, the absorbed dose per voxel is computed as follows:

$$D_{i,j}(E) = I_{i-1,j}(E)\left[1 - \exp\left(-\mu_{i,j}(E)\Delta x\right)\right] \cdot \frac{E}{\Delta m}, \tag{14}$$

where $I_{i-1,j}$ and $I_{i,j}$ represent the number of photons before entering and after exiting the voxel, respectively. The parameter $\mu_{i,j}$ is the linear attenuation coefficient of the voxel, and Δm

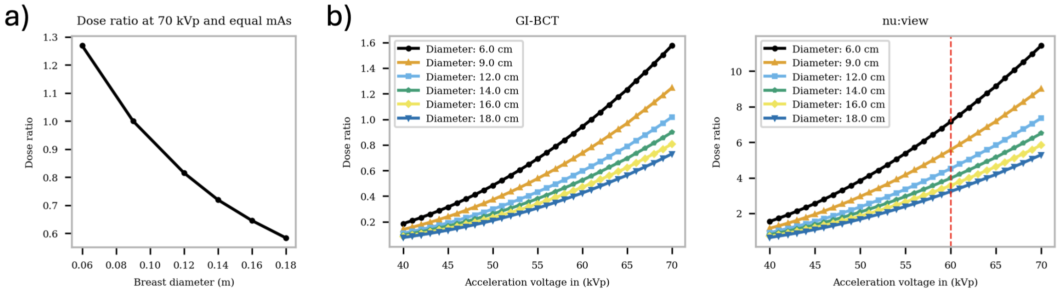

Figure S-4: a) Dose ratio to a 9 cm breast for equal acceleration voltage and charge. b) Dose ratio compared to the mean absorbed dose for a 9 cm breast on the system in [3] for different system geometries and acceleration voltages. Note, that as described in [4] the nu:view system is operated at 60 kVp.

is the mass of that voxel. In this work, the phantom was modeled using the ICRU 44 database for breast tissue [6]. This calculation is repeated for each energy E in the spectrum, using an energy step size of $\Delta E = 0.5\,\text{keV}$. The resulting dose distribution is shown in Fig. S-3b. Since the sample is rotationally symmetric, the full dose distribution for a CT scan can be obtained by summing the rotated volumes:

$$D_{total} = \sum_{m=1}^{M} R_{\theta_m}(D_0), \tag{15}$$

where R_{θ_m} is a rotation transformation around the center of the sample by an angle θ_m and D_0 is the calculated dose distribution for a single angle as in Fig. S-3b. From D_{total} the mean absorbed dose to the sample is calculated as follows [7]:

$$D_{mean} = \frac{\sum_{i\in\Omega} D_{total_i} \Delta m}{\sum_{i\in\Omega} \Delta m}, \tag{16}$$

where Ω is the region inside the sample. To extend the calculated dose values to different breast diameters, geometries, and acceleration voltages, the following procedure was applied. First, the acceleration voltage T and the charge (i.e., the product of exposure time and current, q) were kept constant, while only the breast diameter was varied. The simulation results are shown in Fig. S-3c. The reference dose measurement is taken from Rawlik *et al.*, which reports a mean absorbed dose of 22 mGy for a 9 cm breast at charge $q = 3000\,\text{mAs}$ and acceleration voltage $T = 70\,\text{kVp}$, using the geometry described in Tab. 2 of the main text. Based on this reference, all dose values are scaled to the 9 cm dose value, establishing it as the reference point. The resulting dose ratio is shown in Fig. S-4a, where an expected decrease in mean absorbed dose is observed with increasing breast diameter.

These calculations serve as a reference for all other geometries and acceleration voltages. In the next step, the calculations are adapted for different geometries while keeping the charge constant. For each breast diameter, the mean absorbed dose is then computed for varying acceleration voltages. Based on this, the dose ratio is determined by comparing the dose for equal charge and breast diameter to the reference system from Rawlik *et al.* Finally, using the dose ratio for various breast diameters in the system from Rawlik et al. and the ratio for a newer geometry and acceleration voltage with equal breast diameter, the total dose ratio is calculated. This ratio compares the dose for equal charge to the mean absorbed dose reported by Rawlik *et al.* for a 9 cm breast. The results are shown in Fig. S-4b. Note that the nu:view system does not include gratings, which leads to at least a twofold increase in dose for equal charge.

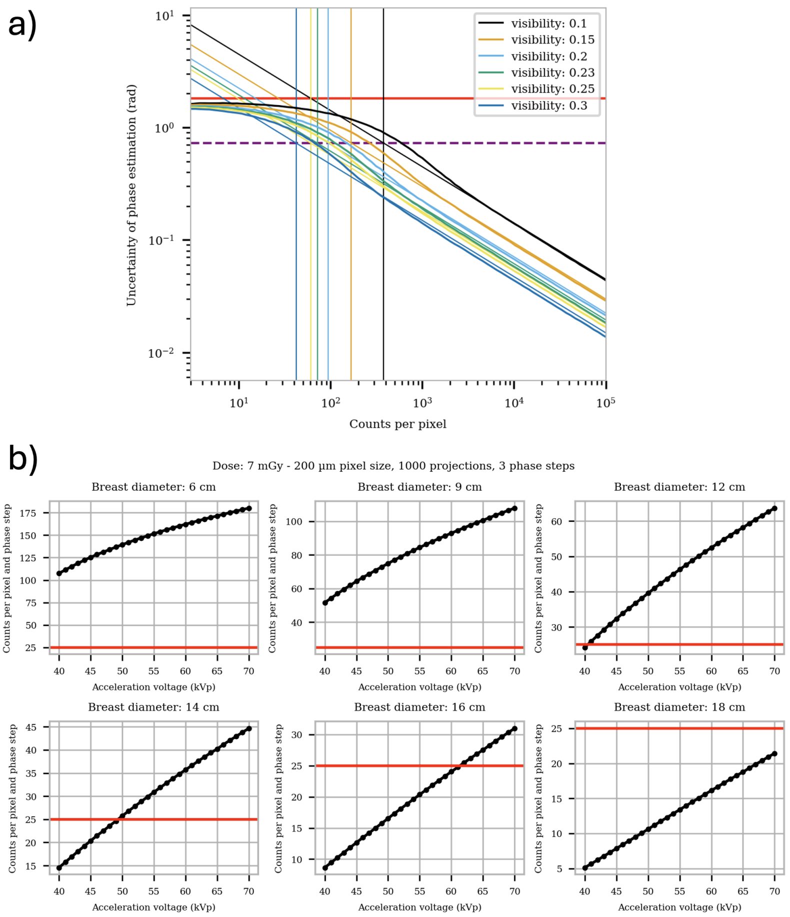

Figure S-5: a) Simulated uncertainty in phase estimation as a function of photon counts per phase step for a single pixel. The figure follows the rationale described in [8]. b) Photon counts per pixel in the new GI-BCT system for different acceleration voltages and breast diameters at 7 mGy for 3 phase steps. The red line indicates the minimum count required to achieve an uncertainty lower than $\sigma_{Max}/2.5$.

5 Photon statistics constraint for phase retrieval

For low photon statistics, Gaussian error propagation is no longer valid, and the noise behavior deviates from the expected trend defined in Eq. 8 from the main text. To prevent information loss, the phase uncertainty should remain below σ_{Max} to avoid cupping artifacts in the reconstructed phase image. Empirically the upper limit in uncertainty was set to $\sigma_{Max}/2.5$ as information could still be retrieved in the simulations. With the simulated visibility (including penalties) around 23 %, at least 75 photon counts per pixel are required. The number of photons per pixel strongly depends on the interferometer's visibility, with higher visibility reducing the required photon count. At low doses, statistical limitations become significant, pushing noise propagation beyond the Gaussian error regime. Using the metric defined in Eq. 8 (main text) and the results from Fig. 3 (main text), the optimal acceleration voltage falls within the range of 40–50 kVp. According to [9], the breast diameter exceeds 16 cm mostly near the chest wall. Given that most of the sample remains below 16 cm in diameter, it is crucial that accurate signal retrieval can be achieved below that diameter, leading to a minimum acceleration voltage of 50 kVp, as shown for the 14 cm breast diameter in Fig. S-5. As breast diameter increases, higher acceleration voltages become necessary. This comes at the cost of reduced refraction sensitivity.

6 Noise power spectrum calculations

Since noise behavior differs between attenuation and refraction (attenuation noise being more prominent at higher frequencies and refraction noise at lower frequencies), a suitable fusion kernel is determined by comparing their respective Noise Power Spectra (NPS). The NPS is computed following the method described in [10], which is briefly recapitulated here. A uniform patch is selected from the images, and the NPS is calculated as follows:

$$NPS(f_i) = \frac{v_x v_y}{N_x N_y} FT\{W(I - \overline{I})\}, \tag{17}$$

where I is the image patch, $\overline{I}$ is its mean intensity, and W is a Hann window function applied to enhance signal clarity. The term FT represents the Fourier transform, while v_x and v_y denote the voxel sizes in the x and y directions, respectively. The parameters N_x and N_y correspond to the number of pixels in the x and y directions. Finally, to simplify the 2D NPS calculation, an azimuthal average is performed, reducing it to a 1D spectrum.

Figure S-6a shows the NPS for refraction and attenuation at various dose levels for a 12 cm sample. The results indicate that refraction exhibits the highest NPS at low frequencies, whereas attenuation dominates at high frequencies. By computing the ratio of the NPS in refraction to that in attenuation, it is possible to determine the frequency at which refraction has lower noise than attenuation (see Fig. S-6b). Using this cutoff frequency, the kernel size for a Gaussian filter is defined as:

$$\sigma_{\text{Fusion}} = \frac{1}{2\pi f_{\text{cutoff}} v_x}, \tag{18}$$

where f_{cutoff} is the frequency at which refraction exhibits a lower NPS than attenuation, and v_x is the voxel size. Figure S-6c further illustrates that the fused signal, obtained using the kernel derived from the NPS ratio, results in an overall lower NPS.

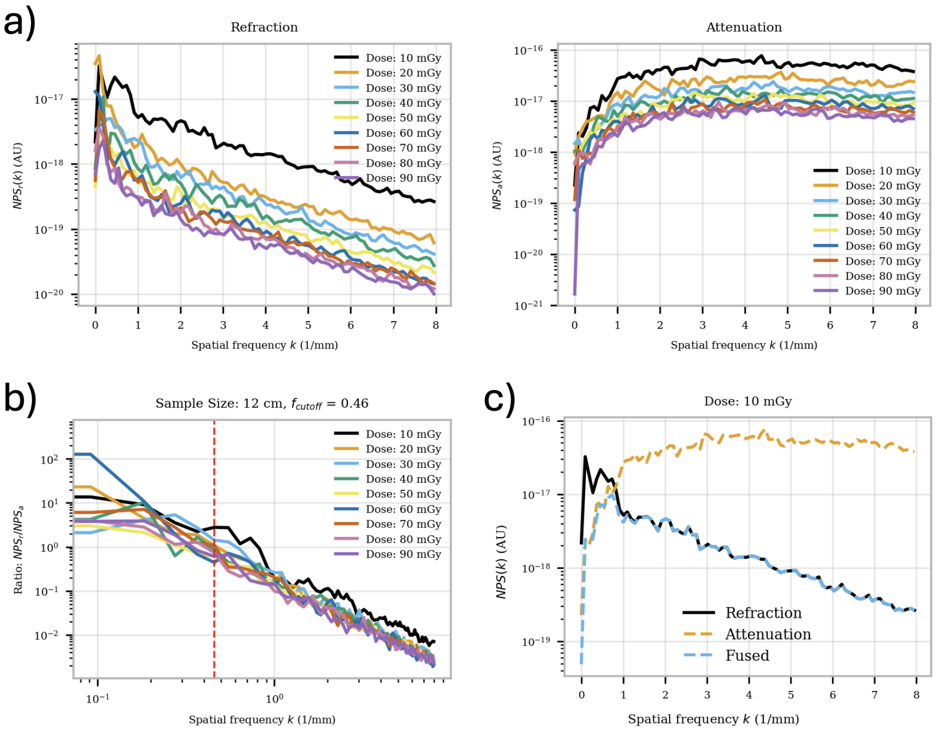

Figure S-6: a) NPS for refraction and attenuation at various dose levels for a 12 cm sample. b) Ratio of the NPS between refraction and attenuation. The marked frequency indicates the threshold where refraction exhibits a lower NPS than attenuation. This frequency is used to determine the kernel size for the Gaussian filter. c) NPS of the fused signal, showing that attenuation dominates at low frequencies while refraction contributes at higher frequencies.

7 Modulation transfer function reduction on the differential phase contrast signal

In [11], the reduction in the modulation transfer function (MTF) of the refraction signal compared to the absorption image was investigated. The study identified that this reduction originates from the destructive superposition of signals from the $+1$ and -1 diffraction orders of the π-shifting G1 grating. To account for this effect, a penalty is applied to the differential phase contrast signal by smoothing it prior to noise addition, using a Gaussian kernel. This correction is necessary because, in the in-silico simulation of the anthropomorphic phantom, no diffraction orders are considered. Thus, in a noise-free scenario, the MTF should be identical for absorption and refraction.

The kernel size is estimated using a wave-propagation simulation [2] of the new system, by projection of a 3 mm graphite rod submerged in water. Water is used to suppress phase-wrapping artifacts. Simulated projections of both absorption and refraction are reconstructed using ASTRA toolbox [12] with a Ram-Lak and Hilbert filter, respectively.

The edge transfer function (ETF) is then computed from the reconstructed images, following the method in [13], by calculating the azimuthal average around the rod to derive the radial profile. The derivative of the ETF is then used to compute the task-transfer function (TTF) (similar to the MTF but is more dependent on the object's contrast and background noise level). The resulting TTF curves are shown in Fig. S-7, where it can be observed that the resolution at 10 % TTF is higher for absorption than for refraction. Therefore, to match the 10 % TTF resolution of refraction, the absorption image must be filtered using a Gaussian kernel.

The Fourier transform of a Gaussian function normalized to unit area is given as:

$$\mathrm{MTF}(f) = \exp\left(-2\pi^2\sigma^2 f^2\right), \tag{19}$$

where σ is the standard deviation of the Gaussian function and f the spatial frequency. Since σ is dependent on the pixel size, the MTF is expressed in dependence of the full width at half maximum (FWHM) as:

$$\mathrm{MTF}(f) = \exp\left(-\frac{(\pi \cdot \mathrm{FWHM} \cdot f)^2}{4\ln 2}\right). \tag{20}$$

From Fig. S-7, it can be seen that the 10 % TTF for absorption is at 6.3 lp/mm and for refraction at 4.4 lp/mm. The goal is to determine the FWHM of the Gaussian blur such that the TTFs match at 10 % TTF. This is done by:

$$\mathrm{TTF}_{new}(4.4) = \mathrm{TTF}_{abs}(4.4) \cdot \mathrm{MTF}_{blurr}(4.4) = 0.1 \tag{21}$$

$$\mathrm{MTF}_{blur}(4.4) = \frac{0.1}{\mathrm{TTF}_{abs}(4.4)} \tag{22}$$

$$\mathrm{FWHM}_{blur} = \sqrt{\frac{-4\ln(2)\ln(\mathrm{MTF}_{blur}(4))}{16\pi}} \tag{23}$$

Assuming a Gaussian form for the TTFs, the resulting FWHM of the kernel required to reduce the absorption image resolution from 6.3 lp/mm to 4.4 lp/mm is approximately 0.124 mm. From this, the corresponding σ in dependence of the voxel size can be calculated and applied.

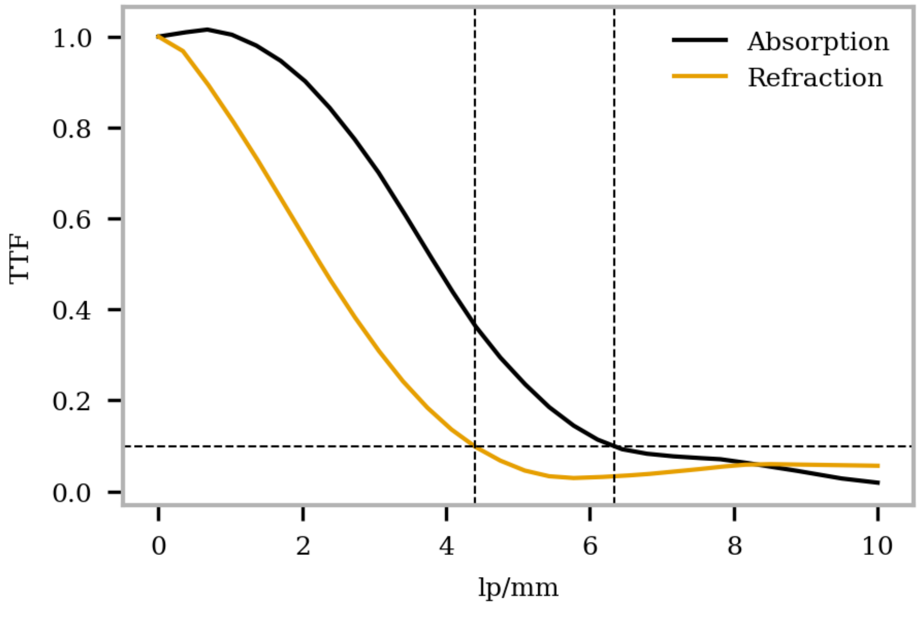

Figure S-7: Task transfer function of a 3 mm graphite rod in water, retrieved from wave-propagation-based simulated projections and reconstructions using ASTRA. The absorption signal shows a slight increase at lower frequencies due to the edge enhancement introduced by the simulation, since the model does not consider any noise.

8 Refraction and Absorption Comparison

In [14], a GI-based breast CT scanner was developed using an optimization metric that focused on maximizing the contrast-to-noise ratio (CNR) gain from phase contrast over attenuation.

This approach led to the construction of a first prototype system at ETH Zürich and the Paul Scherrer Institute. Their study showed that the CNR ratio improves with increasing acceleration voltage; however, this also imposes more stringent and technically challenging requirements on grating fabrication.

In contrast, the optimization metric proposed in this work targets the cumulative CNR, considering both phase and attenuation signals. This leads to improved image quality at significantly lower acceleration voltages, which in turn facilitates grating fabrication. The resulting system demonstrates a notable gain in image quality compared to the previous prototype, primarily due to enhanced refraction signal. This improvement originates from three main factors:

- Increased system length, which enhances the refraction signal as described by Eq. 13.
- Larger refraction angles at lower energies, which inherently increase the refraction signal.
- Lower acceleration voltage, which allows the use of shallower gratings.

The improvement in the refraction signal can visually be seen in Fig. S-8, whereas absorption images look similar (Fig. S-9).

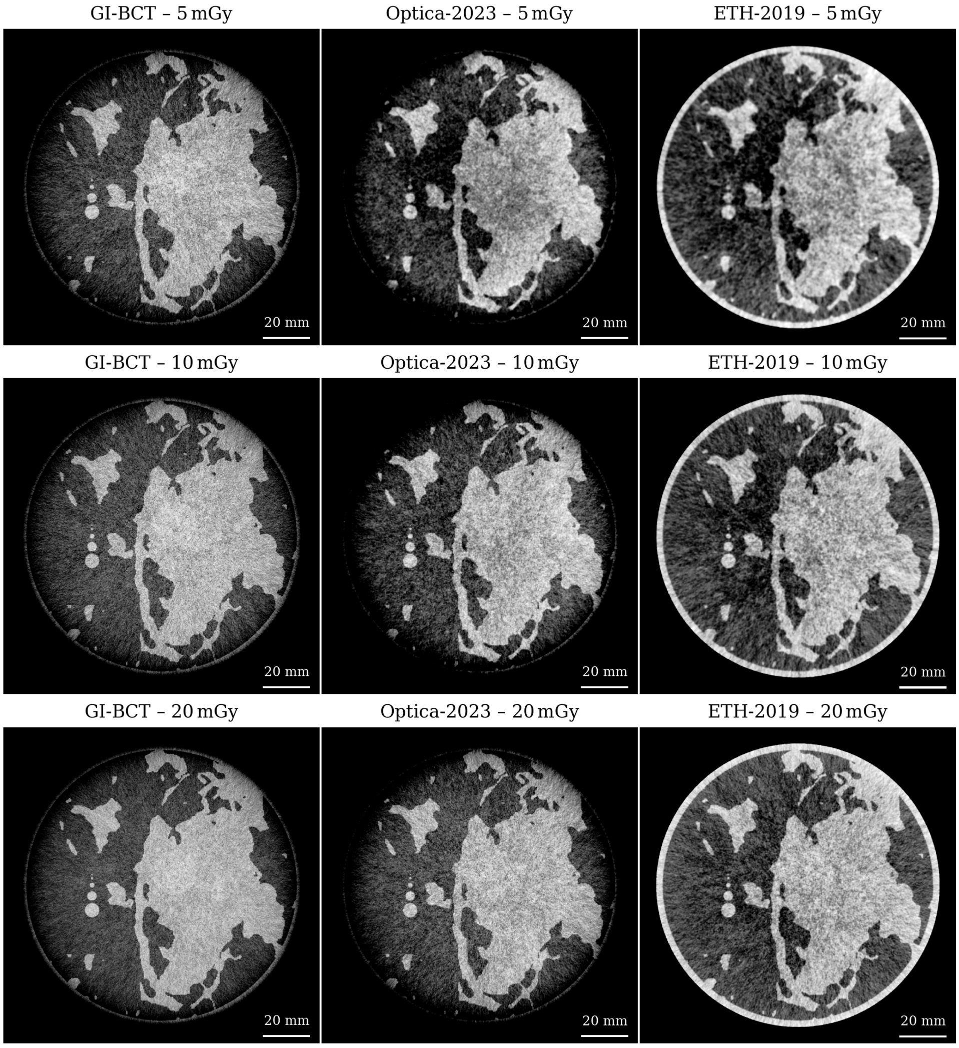

Figure S-8: Refraction reconstructions of the newly optimized system and the GI-system designed for higher CNR ratio between phase and attenuation [3, 14]. The images were Gaussian filtered with kernel sizes optimized for achieving a CNR of 5 between adipose and fibroglandular tissue.

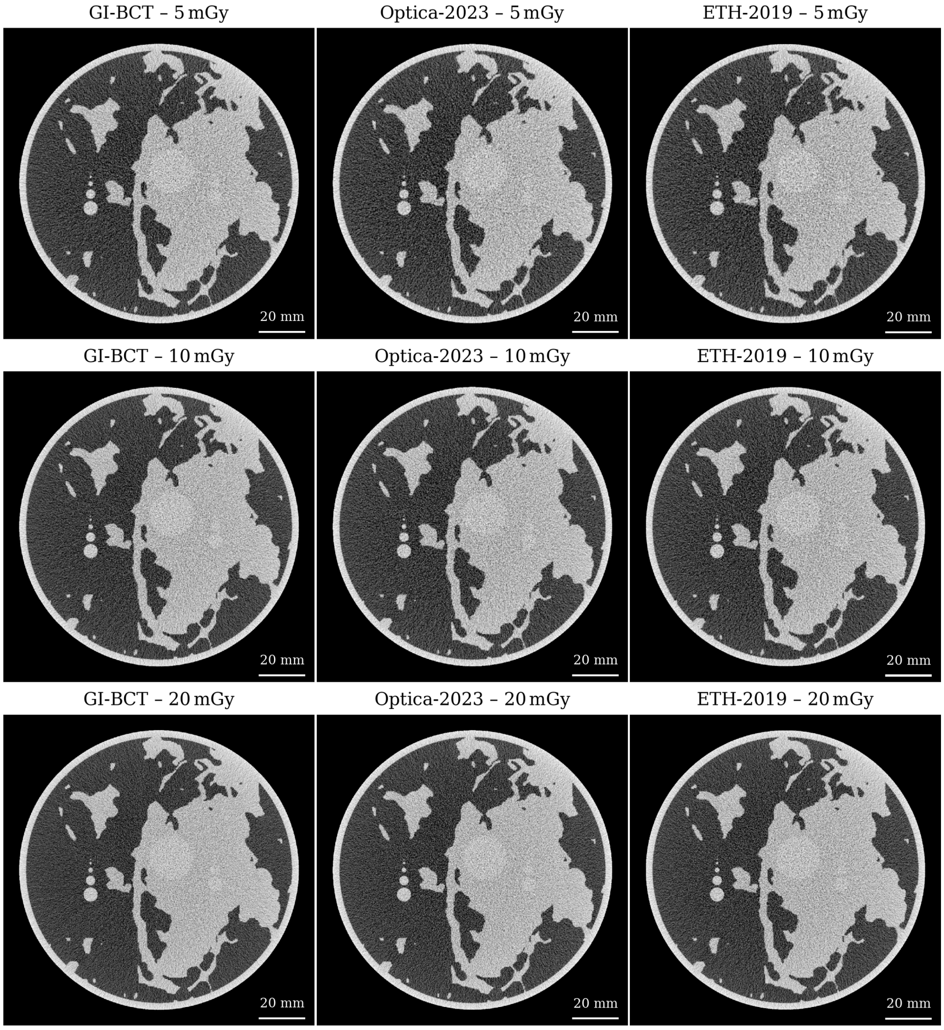

Figure S-9: Absorption reconstructions of the newly optimized system and the GI-system designed for higher CNR ratio between phase and attenuation [3, 14]. The images were Gaussian filtered with kernel sizes optimized for achieving a CNR of 5 between adipose and fibroglandular tissue.

References

[1] T Thuering and M Stampanoni. “Performance and optimization of X-ray grating interferometry”. In: *Philosophical Transactions of the Royal Society A: Mathematical, Physical and Engineering Sciences* 372.2010 (2014). ISSN: 1364503X. DOI: 10.1098/rsta.2013.0027.

[2] Simon Spindler et al. “Simulation framework for X-ray grating interferometry optimization”. In: *Optics Express* 33.1 (Jan. 2025), p. 1345. ISSN: 1094-4087. DOI: 10.1364/OE.543500. URL: https://opg.optica.org/abstract.cfm?URI=oe-33-1-1345.

[3] Michał Rawlik et al. “Increased dose efficiency of breast CT with grating interferometry”. In: *Optica* 10.7 (July 2023), p. 938. ISSN: 2334-2536. DOI: 10.1364/OPTICA.487795. URL: https://opg.optica.org/abstract.cfm?URI=optica-10-7-938.

[4] Sojin Shim et al. “Radiation dose estimates based on Monte Carlo simulation for spiral breast computed tomography imaging in a large cohort of patients”. In: *Medical Physics* 50.4 (Apr. 2023), pp. 2417–2428. ISSN: 0094-2405. DOI: 10.1002/mp.16211. URL: https://aapm.onlinelibrary.wiley.com/doi/10.1002/mp.16211.

[5] Gavin Poludniowski et al. “Technical Note: SpekPy v2.0—a software toolkit for modeling x-ray tube spectra”. In: *Medical Physics* 48.7 (July 2021), pp. 3630–3637. ISSN: 24734209. DOI: 10.1002/MP.14945.

[6] MD Bethesda. “Tissue substitutes in radiation dosimetry and measurement”. In: *International Commission on Radiation and Measurements* (1989).

[7] Johan Gustafsson et al. “Averaging of absorbed doses: How matter matters”. In: *Medical Physics* 50.10 (Oct. 2023), pp. 6600–6613. ISSN: 2473-4209. DOI: 10.1002/MP.16528. URL: https://onlinelibrary.wiley.com/doi/full/10.1002/mp.16528https://onlinelibrary.wiley.com/doi/abs/10.1002/mp.16528https://aapm.onlinelibrary.wiley.com/doi/10.1002/mp.16528.

[8] Rainer Raupach and Thomas G Flohr. “Analytical evaluation of the signal and noise propagation in x-ray differential phase-contrast computed tomography”. In: *Physics in Medicine and Biology* 56.7 (Apr. 2011), pp. 2219–2244. ISSN: 0031-9155. DOI: 10.1088/0031-9155/56/7/020. URL: https://iopscience.iop.org/article/10.1088/0031-9155/56/7/020.

[9] Shih Ying Huang et al. “The characterization of breast anatomical metrics using dedicated breast CT”. In: *Medical Physics* 38.4 (2011), pp. 2180–2191. ISSN: 00942405. DOI: 10.1118/1.3567147.

[10] Steven Dolly et al. “Practical considerations for noise power spectra estimation for clinical CT scanners”. In: *Journal of Applied Clinical Medical Physics* 17.3 (May 2016), pp. 392–407. ISSN: 1526-9914. DOI: 10.1120/JACMP.V17I3.5841. URL: https://onlinelibrary.wiley.com/doi/full/10.1120/jacmp.v17i3.5841https://onlinelibrary.wiley.com/doi/abs/10.1120/jacmp.v17i3.5841https://aapm.onlinelibrary.wiley.com/doi/10.1120/jacmp.v17i3.5841.

[11] Yuhang Tan et al. “On the origin of MTF reduction in grating-based x-ray differential phase contrast CT imaging”. In: *Medical Physics* 52.3 (Mar. 2025), pp. 1546–1555. ISSN: 24734209. DOI: 10.1002/MP.17593.

[12] Wim van Aarle et al. “Fast and flexible X-ray tomography using the ASTRA toolbox”. In: *Optics Express* 24.22 (Oct. 2016), p. 25129. ISSN: 1094-4087. DOI: 10.1364/OE.24.025129.

[13] Ehsan Samei et al. "Performance evaluation of computed tomography systems: Summary of AAPM Task Group 233". In: *Medical Physics* 46.11 (Nov. 2019), e735–e756. ISSN: 24734209. DOI: 10.1002/MP.13763;WEBSITE:WEBSITE:AAPM;PAGE:STRING:ARTICLE/CHAPTER. URL: /doi/pdf/10.1002/mp.13763https://onlinelibrary.wiley.com/doi/abs/10.1002/mp.13763https://aapm.onlinelibrary.wiley.com/doi/10.1002/mp.13763.

[14] Maria Büchner. "Towards the development of an X-Ray phase contrast breast CT scanner". PhD thesis. ETH Zürich, 2019.
